# Retrospective clinical case series study in 2017 identifies *Plasmodium knowlesi* as most frequent *Plasmodium* species in returning travellers from Thailand to Germany

**DOI:** 10.2807/1560-7917.ES.2018.23.29.1700619

**Published:** 2018-07-19

**Authors:** Guenter Froeschl, Hans Dieter Nothdurft, Frank von Sonnenburg, Gisela Bretzel, Roman Polanetz, Inge Kroidl, Michael Seilmaier, Hans Martin Orth, Sabine Jordan, Peter Kremsner, Sabine Vygen-Bonnet, Michael Pritsch, Michael Hoelscher, Camilla Rothe

**Affiliations:** 1 Division of Infectious Diseases and Tropical Medicine, Medical Center of the University of Munich (LMU), Munich, Germany; 2 German Center for Infection Research (DZIF), Partner Site Munich, Munich, Germany; 3 Klinikum München Schwabing, Munich, Germany; 4 Klinik für Gastroenterologie, Hepatologie und Infektiologie, Universitätsklinikum Düsseldorf, Düsseldorf, Germany; 5 University Medical Center Hamburg-Eppendorf, 1st Medical Department, Division of Tropical Medicine and Infectious Diseases, Hamburg, Germany; 6 Institut für Tropenmedizin, Reisemedizin und Humanparasitologie des Universitätsklinikums Tübingen, Tübingen, Germany; 7 Robert Koch-Institute, Department of Infectious Disease Epidemiology, Unit of Gastrointestinal Infections, Zoonoses and Tropical Infections, Berlin, Germany

**Keywords:** *Plasmodium knowlesi*, malaria, rapid diagnostic test, travel, Thailand, Germany

## Abstract

Febrile illnesses are common in travellers returning from south-east Asia. However, malaria is a rare diagnosis in this population. A series of *Plasmodium knowlesi* infections was noted in German travellers returning from Thailand since 2012. Infectious disease and tropical medicine facilities registered by the German Society for Tropical Medicine and International Health were contacted in March 2017, and asked to report previous *P. knowlesi* cases. In addition, surveillance data from the Robert Koch-Institute were analysed. The facilities reported a total of six *P. knowlesi*-positive cases, all were returning travellers from Thailand. The *P. knowlesi*-positive cases made up 6/9 of all diagnosed malaria cases imported from Thailand in the time period 2012 to 2017. In 4/5 of cases where a malaria rapid diagnostic test had been applied it revealed a negative result. *P. knowlesi* is an important differential diagnosis in travellers returning from south-east Asia with itineraries that include Thailand. This study highlights the importance of this *Plasmodium* species in this patient subgroup. Whenever malaria is suspected in a returning traveller from Thailand, *P. knowlesi* should be taken into consideration and a differential PCR be executed as currently the unequivocal diagnosis of *P. knowlesi* is based on nuclear amplification techniques.

## Background

Germany is currently the second leading country worldwide in numbers of international travels with about 84 million international travel departures in 2015, most of them for leisure purposes [1]. About one in seven international travellers to overseas from Germany suffers from a medical condition during their travel, and among those affected, about one quarter require some kind of medical assistance [2]. Malaria remains a relevant differential diagnosis in febrile patients arriving from tropical and subtropical areas, with 1,068 imported malaria cases reported in Germany in 2015 [3]. Thailand is a favourite travel destination for German travellers with 761,000 arrivals from Germany in 2015. In the same year, Thailand reported almost 30 million international arrivals in total [4].

Malaria is considered hypoendemic in Thailand with a reported total of 14,755 confirmed cases and 33 deaths in a total population of about 68 million in 2015 (incidence: 21 cases/100,000). However, the estimated true incidence of malaria infections is expected to be much higher at an annual incidence rate of 176 cases per 100,000 population [5].

The German guidelines for malaria prevention in travellers are issued annually by the German Society for Tropical Medicine and International Health (DTG). DTG recommends carrying an anti-malarial standby medication when travelling to Thai regions bordering Cambodia, Laos and Myanmar only, and only if urban settings with a presumably adequate medical infrastructure are left behind [6].

The *Plasmodium* species mentioned in the latest 2016 World Malaria Report as being prevalent in Thailand are *P. falciparum* (41.8% of all specified cases) and *P. vivax* (58.2%) [5]. Previous publications in 2011 and 2015 have indicated occasional cases of *P. knowlesi* infections in the resident population of Thailand [7,8]. In addition, on rare occasions, *P. knowlesi* infections in German travellers returning from Thailand have been reported [9-13]. Published studies, mostly on the basis of individual case reports, from other European countries, such as Finland, France, Spain, Sweden and the Netherlands, have also reported on importations of *P. knowlesi* infections from south-east Asia in returning travellers, indicating the diagnosis of a *P. knowlesi* infection as a generally rare event. The advent of occasional *P. knowlesi* identifications in returning European travellers seems to have started about a decade ago [14-20]. Of note, a recent analysis by the GeoSentinel Network, a global network for surveillance of travel-related illnesses, which included data from 29 countries, only reported three cases between 2003 and 2016, all imported from south-east Asia, whereby the explicit countries of acquisition were not indicated [21].

Malaria caused by *P. knowlesi* is a zoonotic infection endemic to south-east Asia, with a primary reservoir in macaques. The geographical distribution of *P. knowlesi* is therefore linked to the presence of its primary hosts. Humans can be infected occasionally, especially in areas where human settlements are advancing into habitats of the reservoir host, namely forested areas. Investigations on archived blood samples in Malaysia have revealed that the large majority of cases previously classified by microscopy as *P. malariae* infections were in fact *P. knowlesi* infections [22]. The parasite species has a short replication cycle of only 24 hours and can thus rapidly generate high parasite loads [23]. More frequently than in infections with *P. malariae*, *P. vivax* and *P. ovale*, *P. knowlesi* causes severe courses of disease that resemble *P. falciparum* malaria. In particular, respiratory and renal complications have been reported [16].

Upon noticing a series of *P. knowlesi* cases in people returning from Thailand to Germany, this study was conceived in order to investigate the relevance of this parasite as a causative agent for febrile conditions in such travellers.

## Methods

### Questionnaire

A digital questionnaire was compiled in the format of a case report file (Microsoft Word). For each reported *P. knowlesi* case, one questionnaire was to be completed by the facility (as described below) that identified the case. Questions included age, sex, purpose of travel, travel itinerary, date of return, date of presentation, clinical presentation, means of diagnosis employed, highest level of care, treatment regimen and clinical course. In addition, details were requested on the total number of malaria cases in the respective facility for the respective reporting year in patients returning from the same country from which the reported *P. knowlesi* case had returned.

### Data sources

The DTG is a reference institution in the field of travel medicine in Germany with annual publications on malaria risk profiles for worldwide travel destinations and guidelines on prevention and treatment of malaria. The society lists all major infectious diseases and tropical medicine facilities in Germany. The complete list of 17 facilities was contacted on 16 March 2017 with an invitation to participate in the study by reporting whether *P. knowlesi* cases had been diagnosed previously at each respective facility, and if so, to complete one questionnaire per *P. knowlesi* case. Furthermore, corresponding authors of already published cases (who were found as further described) were directly contacted. The last completed form was returned on 23 May 2017.

In addition, a literature search of articles in English language in PubMed using the keywords ‘knowlesi’ AND ‘Germany’ was conducted. Malaria in Germany is mandatorily reported to the national public health institute (the Robert Koch-Institute; RKI). The archive and surveillance data of the RKI were searched to potentially identify additional cases [24].

## Results

### Case finding

In total 17 facilities were contacted, including the facility of the study coordinators. Sixteen of the 17 facilities replied and provided completed questionnaires if cases had been observed. Eleven facilities indicated never having diagnosed any case of *P. knowlesi* infections. Four facilities reported one case each and one facility reported two respective cases, resulting in a total of six *P. knowlesi* cases. The earliest case ever reported in Germany was in 2012 and all six cases had travelled to Thailand. A literature search revealed only cases who were also reported directly by the participating facilities [9-13]. 

An investigation of the archive and the surveillance data of the RKI provided a total of nine notified malaria cases across all *Plasmodium* species that were imported from Thailand to Germany in the time period 2012 to 2017 as denominator. As part of these nine, all six *P. knowlesi* cases who were reported by the specialised facilities through this study could also be retrieved in the RKI surveillance data. In addition, one *P. knowlesi* case with missing information on travel purpose, destination and probable country of infection was revealed by the surveillance data; this case could not be included in the analysis.

### Epidemiological and demographic description of cases

The reported six cases comprised four males and two females, the age range was 42 to 73 years and all cases were German citizens. The earliest case was from 2012 and had travelled to Thailand. Six further cases of *P. knowlesi* infections subsequently occurred, five of which had also travelled to Thailand and one with travel information missing, as mentioned above. Cases are hereafter referred to by case identification numbers, as outlined in the Table.

**Table:** Cases of *Plasmodium knowlesi* infections reported in Germany, 2012–2017

Case ID	Date of admission	Age in years	Sex	Level of care	RDT	Smear parasitaemia^a^	Nuclear amplification techniques	Treatment
1	January 2012	54	Male	IPD	Not done	0.01%	Sequencing positive^b^	Atovaquone/proguanil
2	January 2013	55	Female	IPD	BinaxNOW, pan-aldolase (T2 band) positive	0.2%	PCR positive^c^	Artesunate followed by Artemether/lumefantrine
3	December 2013	73	Male	ICU	BinaxNOW negative	3%	PCR positive^c^	Quinine/doxycycline
4	December 2014	52	Female	IPD	BinaxNOW negative	1%	PCR positive^c^	Artemether/lumefantrine
5	February 2015	42	Male	IPD	BinaxNOW negative	0.02%	PCR positive^c^	Atovaquone/proguanil
6	January 2017	45	Male	OPD	BinaxNOW negative	0.0002%	PCR positive^c^	Atovaquone/proguanil

ICU: intensive care unit; IPD: in-patient department; OPD: out-patient department; RDT: rapid diagnostic test.

^a^ Percentages of infected erythrocytes are presented.

^b^ Sequencing of small subunit ribosomal RNA, Basic Local Alignment Search Tool analysis of the obtained sequence, yields a 96% match with the respective *P. knowlesi* sequence.

^c^ A PCR with primers specific for *P. knowlesi* was conducted.

Tourism was the main purpose of travel in five of six cases, one case reported to have visited friends and relatives (case 1). All travels took place in the months of November to February.

From a more detailed investigation of the respective travel itineraries, it was noted that all cases had travelled to the southern Thai provinces of Ranong, Phang-Nga and Surat Thani (Figure).

**Figure:**
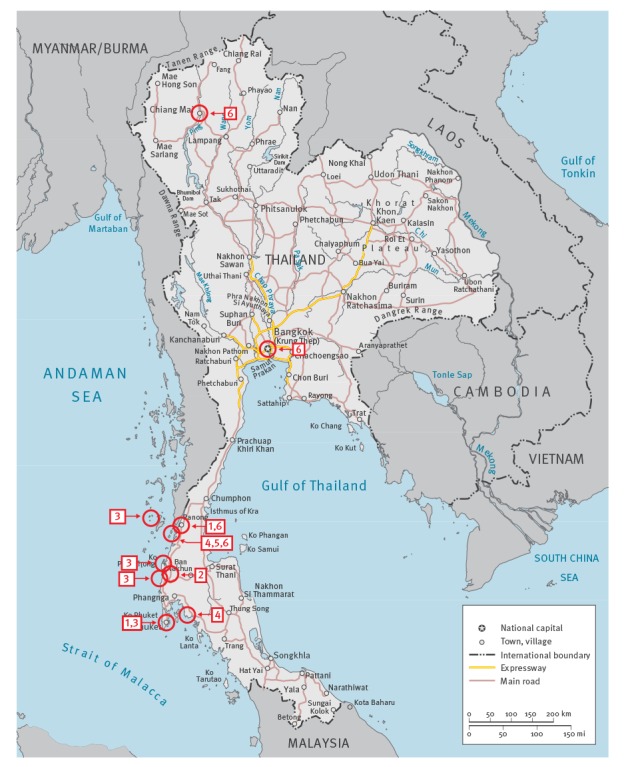
Map of Thailand with travel destinations of cases of *Plasmodium knowlesi* infections, 2012–2017

This map is adapted from a map by the Department of Field Support, Cartographic Section of the United Nations [32]. Encircled in red are locations of stay as indicated by the reported cases. Numbers in arrowed boxes are indicating case IDs as outlined in the Table.

It has to be pointed out that the island of (Little) Koh Chang in the Andaman Sea, which should not be confused with the larger island of Koh Chang in the Gulf of Thailand, was mentioned as a destination by three of the travellers (cases 4,5,6), and for all three this island was the location with a considerably longer duration of stay. Only one patient (case 3) also travelled outside Thailand during the itinerary; however, his destination in neighbouring Myanmar was the island of Macleod, which lies in the immediate vicinity of Ranong Province. In addition to the above named provinces, patients mentioned Bangkok as city of entry including just a brief stop-over, and one patient (case 6) also travelled to the northern city of Chiang Mai before passing the largest part of the stay in the southern province of Ranong (Figure).

### Case clinical presentation, diagnostics and management

All cases presented with fever (temperature ≥ 38.0 °C) as the leading symptom. The lag-time between return from travel and presentation at the facilities was between 1 and 23 days. In all cases a positive blood smear led to the diagnosis of a *Plasmodium* infection. In 5/6 cases, the microscopy result was initially reported as presence of *P. falciparum* or *P. malariae*, but also morphological resemblance to *P. vivax* and *P. ovale* was reported in two respective cases. Co-infections by multiple species were reported in the initial smear results in three of the six cases. The parasite load was in the range between 0.0002% and 3%. The parasite load was particularly low in case 6, where the microscopy result that led to further investigations was based on a single gametocyte in a total of 100 investigated fields at 400 x magnification. In five of the six cases (cases 2–6) a rapid diagnostic test (RDT) for malaria was conducted. All facilities used the BinaxNOW Malaria assay (Alere, Scarborough, Maine, USA). In four of five RDTs conducted the test revealed a negative result. Only one facility reported a positive T2 band (case 2), corresponding to the pan-plasmodial aldolase (Table). According to the BinaxNOW Malaria product specifications a positive T2 band with a negative T1 band is interpreted as a mono- or mixed infection by *P. vivax*, *P. ovale* or *P. malariae*, in the absence of *P. falciparum*. All six reported cases were confirmed to be *P. knowlesi* by nuclear amplification techniques. For case 1 the small subunit ribosomal RNA sequence was amplified and the sequence was further analysed by Basic Local Alignment Search Tool (BLAST). This yielded 96% identitity with sequencing data from *P. knowlesi* [13]. For the other cases a differential PCR with primers for *P. knowlesi* was conducted. Five of the six cases, were hospitalised. In two cases the World Health Organization (WHO) criteria for severe malaria were fulfilled [25]. In one case admission to an intensive care unit was necessary due to severe presentation with renal and respiratory insufficiency, acidosis and reduced vigilance (case 3). This case was at the same time the one with the highest parasitaemia of 3% in this case series. A second case of complicated *P. knowlesi* malaria was treated with renal impairment; however, in this case intensive care was not pursued (case 2). Treatment was in all cases initiated prior to the conclusive diagnosis of *P. knowlesi* infection, and corresponded with the results of initial microscopy. As the applied regimens were assumed to be effective also in *P. knowlesi* infections, no change in treatment regimen had to be executed in any of the cases (Table).

The treatment outcome was considered successful in all cases. The time until improvement of symptoms was indicated as 1, 2 and 3 days after commencement of anti-malarial treatment, for three of the reporting facilities where this information was retrievable from files. No recrudescence was reported in any of the cases.

### Proportion of *Plasmodium knowlesi* cases among malaria cases imported from Thailand to Germany

The five facilities reporting *P. knowlesi* cases were asked to indicate the total number of malaria cases for the same travel destination from which the index case had imported the *P. knowlesi* infection (in all cases Thailand) for the respective year of diagnosis of the *P. knowlesi* case. In total, seven cases of malaria were reported by the facilities in returning travellers from Thailand in the respective years, including the six cases of *P. knowlesi* infections. Only one facility indicated one additional case other than *P. knowlesi* in a returning traveller from Thailand for the year 2014. The proportion of *P. knowlesi* infections among all malaria cases from Thailand in the reporting facilities for the reported years is therefore amounting to 6 of 7. When the total number of nine malaria cases who were reported to the RKI as imported from Thailand to Germany in the time period 2012 to 2017 is taken as denominator, the proportion of *P. knowlesi* infections amounts to 6 of 9 of all imported cases from Thailand. The remaining cases were two *P. vivax* and one *P. falciparum* infection.

## Discussion and conclusions

Malaria remains a very low risk for German travellers to the favourite travel destination Thailand. In 2015, a total of 761,000 travellers from Germany departed for this country. The RKI reported a total number of 1,068 imported malaria cases to Germany for 2015. Only two of these were imported from Thailand [3]. In the entire time period from 2012 to 2017, only a total of nine malaria cases were reported to the national public health institute as imported from Thailand. The facilities participating in this study, which are among the largest facilities in Germany specialised in the field of infectious diseases and tropical medicine, identified six infections that were due to *P. knowlesi*. This species is not reported as prevalent in Thailand in the latest World Malaria Report of 2016 [5]. Given the finding, that in most cases a *Plasmodium* species other than *P. knowlesi* was initially morphologically indicated, and that the common range of anti-malarials applied are also active against *P. knowlesi*, we assume that imported *P. knowlesi* infections may frequently remain undetected. It is of note that resemblance in *P. knowlesi* morphology may be indicative of the stage in replication cycle, as early stages have been reported to rather resemble *P. falciparum*, whereas later stages more frequently show features of *P. malariae* [26].

In addition, more and more non-specialist institutions rely for their primary diagnosis of malaria on RDTs, which are highly sensitive for *P. falciparum* malaria but frequently fail to detect non-falciparum species [27]. In this study, the specialised facilities executed an RDT in five of the six *P. knowlesi* cases. In all cases the RDT used was the BinaxNOW Malaria. Only one test revealed a positive result for the aldolase antigen only. The BinaxNOW has been designed and validated by the manufacturer for *P. falciparum* and *P. vivax* infections only. The T1 band represents the histidine-rich-protein II, which is a specific antigen for *P. falciparum*, whereas the T2 band represents the pan-plasmodial aldolase antigen. For *P. falciparum* the manufacturer indicates an overall sensitivity of 95.3% and a specificity of 94.2%; the test performance is lower in *P. vivax* infections, with an overall sensitivity of 68.9% (specificity 99.8%). The product information by the manufacturer indicates that the RDT is also able to detect *P. ovale* and *P. malariae* but for these species the clinical performance has not been adequately established. *P. knowlesi* is not mentioned in the test specifications [28]. A study conducted in Malaysia reported low sensitivity of RDTs that are based on the pan-plasmodial aldolase for all *Plasmodium* species, with the lowest sensitivity in *P. knowlesi* infections at 23%. Although sensitivity generally increased with parasite load, only 45% of highly parasitaemic patients with a *P. knowlesi* infection (> 10,000 parasites/µL) revealed a positive result [27]. This finding is corroborated by the results of this study where one positive pan-plasmodial aldolase test was found in five patients. RDTs with a pan-plasmodial lactate dehydrogenase component generally seem to perform with a higher sensitivity for all species including *P. knowlesi*, but even there one in four *P. knowlesi* infections remains undetected by the assay [27]. Of note, three of the six cases reported in this study had a low parasitaemia of < 0.1% infected erythrocytes, which contributes to impaired test performance even more.

In settings where malaria detection is depending on RDT performance, a possible case of *P. knowlesi* malaria is likely to remain undetected or to be complicated by late diagnosis. Severe courses of infection in immunologically naive individuals are assumed to be less frequent in *P. knowlesi* than in *P. falciparum* infections, but more frequent than in *P. malariae*, *P. vivax* or *P. ovale*, which corroborates the importance of a valid species differentiation. In this study, two of six cases fulfilled criteria for severe malaria.

Since malaria still is a possible aetiology for febrile conditions in returning travellers with a potentially severe clinical course, the findings of this study suggest that *P. knowlesi* may need to be added to the risk profile of Thailand. The high proportion of *P. knowlesi* infections in the investigated population of this study (6/9) demands that once the diagnosis of a malaria infection is established in a traveller returning from Thailand (or indeed elsewhere from south-east Asia), a differential malaria PCR that comprises *P. knowlesi* should be executed, irrespective of the species differentiation through microscopy, and irrespective of an RDT result. In addition, in cases with a persistent suspicion of a malaria infection but with negative smear and RDT results, a differential PCR comprising *P. knowlesi* should be considered as a means of diagnosis of a possible *P. knowlesi* infection presenting with a low parasitaemia. However, the extremely low incidence of only two cases of malaria across all species in an annual 761,000 travels from Germany to Thailand may provoke a reconsideration of current travel recommendations with regard to malaria prevention, especially in light of reports of inadequate use of anti-malarial standby medication [29]. 

The findings of this study are expected to be of equal relevance to other European countries with comparable tourism profiles of their citizens. As we previously highlighted, identification of *P. knowlesi* remains currently a rare event for countries such as Finland, Sweden, Spain and France. However, as the overall incidence of *Plasmodium* infections across all species in European travellers returning from south-east Asia is largely declining [30] the consideration of *P. knowlesi* infections becomes important based on our and earlier findings. 

There are some limitations to this study. A selection bias could have occurred, as only infectious disease and tropical medicine facilities registered by the DTG were contacted. A relevant number of cases of malaria are detected outside these facilities in Germany. In addition, the authors suspect a number of unrecognised — or more specifically misclassified — cases of *P. knowlesi* infections, as in many cases of febrile illnesses in returning travellers from Thailand the necessary differential PCR will not have been executed. The authors are convinced that a number of the cases, who were previously classified as infections by especially *P. malariae* and *P. falciparum* from south-east Asia, were actually infected by *P. knowlesi*.

The importance of considering *P. knowlesi* in malaria control efforts in south-east Asia has been pointed out in a recent publication by Barber et al. [31]. Taking travellers as sentinels, our cases series may highlight an, as yet, under-addressed public health problem in some parts of Thailand. Further PCR-based studies in targeted areas of Thailand should be of public health interest to find out which share *P. knowlesi* infection takes in human as well as in simian malaria.
